# Single‐Molecule Insight Into α‐Synuclein Fibril Structure and Mechanics Modulated by Chemical Compounds

**DOI:** 10.1002/advs.202416721

**Published:** 2025-02-14

**Authors:** Xiang Li, Lulu Bi, Shenqing Zhang, Qianhui Xu, Wencheng Xia, Youqi Tao, Shaojuan Wu, Yanan Li, Weidong Le, Wenyan Kang, Dan Li, Bo Sun, Cong Liu

**Affiliations:** ^1^ Bio‐X Institutes Key Laboratory for the Genetics of Developmental and Neuropsychiatric Disorders (Ministry of Education) Shanghai Jiao Tong University Shanghai 200030 China; ^2^ Zhangjiang Institute for Advanced Study Shanghai Jiao Tong University Shanghai 201203 China; ^3^ School of Life Science and Technology ShanghaiTech University Shanghai 201210 China; ^4^ Interdisciplinary Research Center on Biology and Chemistry Shanghai Institute of Organic Chemistry Chinese Academy of Sciences Shanghai 201210 China; ^5^ University of the Chinese Academy of Sciences Chinese Academy of Sciences Beijing 100049 China; ^6^ Shanghai University of Medicine and Health Sciences Affiliated Zhoupu Hospital Shanghai 201318 China; ^7^ Department of Neurology and Institute of Neurology Ruijin Hospital Shanghai Jiao Tong University School of Medicine Shanghai 200025 China; ^8^ State Key Laboratory of Chemical Biology Shanghai Institute of Organic Chemistry Chinese Academy of Sciences Shanghai 200032 China; ^9^ Shanghai Academy of Natural Sciences (SANS) Fudan University Shanghai 200433 China

**Keywords:** chemical compounds, optical tweezers, parkinson's disease, single molecule, α‐synuclein fibril

## Abstract

α‐Syn fibrils, a key pathological hallmark of Parkinson's disease, is closely associated with disease initiation and progression. Several small molecules are found to bind or dissolve α‐syn fibrils, offering potential therapeutic applications. Here, an innovative optical tweezers‐based, fluorescence‐combined approach is developed to probe the mechanical characteristics of α‐syn fibrils at the single‐molecule level. When subjected to axial stretching, local deformation within α‐syn fibrils appeared at forces above 50 pN. These structural alternations occurred stepwise and are irreversible, suggesting unfolding of individual α‐syn molecules or subdomains. Additionally, α‐syn fibrils exhibits high heterogeneity in lateral disruption, with rupture force ranging from 50 to 500 pN. The impact of different compounds on the structure and mechanical features of α‐syn fibrils is further examined. Notably, epigallocatechin gallate (EGCG) generally attenuates the rupture force of fibrils by wedging into the N‐terminal polar groove and induces fibril dissociation. Conversely, copper chlorophyllin A (CCA) attaches to four different sites wrapping around the fibril core, reinforcing the stability of the fibril against rupture forces. The work offers an effective method for characterizing single‐fibril properties and bridges compound‐induced structural alternations with mechanical response. These insights are valuable for understanding amyloid fibril mechanics and their regulation by small molecules.

## Graphical abstract

This work developed an innovative method combining optical tweezers and fluorescence microscopy to study the mechanics of individual α‐synuclein fibrils, a hallmark of Parkinson's disease. At the single‐fibril level, α‐syn fibrils exhibit rigidity and heterogeneity in disruption and deformation. By integrating cryo‐EM, compound‐induced structural changes are linked to mechanical responses, offering insights into fibril mechanics and their regulation by small molecules.

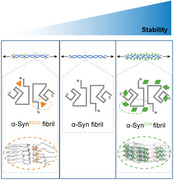

## 1 Introduction

Abnormal aggregation and fibrillation of α‐synuclein (α‐syn) are closely linked to the onset and progression of various neurodegenerative diseases (NDs) such as Parkinson's Disease (PD), Multiple System Atrophy (MSA) and Dementia with Lewy Bodies (DLB).^[^ ^1^, ^2^, ^3^, ^4^ ^]^ α‐Syn fibril is also recognized as the key pathological hallmark of these diseases.^[^ ^5^, ^6^, ^7^, ^8^ ^]^ Hence, targeting α‐syn fibrils is crucial for therapeutic interventions and early disease diagnosis. These fibrils are involved in a wide range of pathological processes, including cell‐to‐cell transmission, cellular membrane disruption through mechanical forces, and the triggering of neuroinflammation.^[^ ^9^, ^10^, ^11^, ^12^, ^13^, ^14^ ^]^ In addition, the superior properties of α‐syn fibrils make them potential building blocks for protein‐based nanomaterials.^[^ ^15^, ^16^ ^]^ Recently, detailed cryo‐electron microscopy (cryo‐EM) studies have provided extensive information on the atomic structures of pathological α‐syn fibrils calculated by averaging thousands of fibrils in a frozen and static state, significantly advancing our understanding of their assembly process.^[^ ^17^, ^18^, ^19^, ^20^, ^21^, ^22^, ^23^, ^24^ ^]^ Nevertheless, examining the structure and mechanical characteristics of α‐syn fibrils in solution at a single‐fibril level is still challenging. Consequently, the mechanics regarding the maintenance and disruption of the α‐syn fibril structure are not fully understood.

Given the significant role of α‐syn fibrils in various NDs, extensive research has been conducted to develop chemicals for either binding to the fibrils for diagnosis or disrupting them for potential treatment. Various compounds, including epigallocatechin gallate (EGCG), Curcumin, and others, have been identified for their strong ability to break down α‐syn fibrils.^[^ ^25^, ^26^, ^27^, ^28^ ^]^ Meanwhile, compounds like BF227 and copper chlorophyllin A (CCA) demonstrate a high affinity for binding across multiple sites on the fibril core of α‐syn.^[^ ^29^ ^]^ However, the specific impact of these compounds on α‐syn at the single‐fibril level remains poorly understood. This knowledge gap limits our comprehension of the interaction between fibrils and compounds and the development of effective therapeutic and diagnostic agents.

In this work, we established an optical tweezers (OT)‐based approach to investigate the mechanical properties of α‐syn fibrils in solution at a single‐fibril level, wherein its morphology can be fluorescently monitored at the same time. Examining the elastic response revealed a rigid biopolymer of α‐syn fibrils, with a persistence length of a few micrometers and an axial elastic modulus of thousands of piconewtons. Interestingly, over 50 pN, α‐syn fibrils randomly displayed stepwise increments in length before fibril rupture. Quantitative analysis of the corresponding fluorescence imaging and the step size attributes these sudden changes to the local structural deformation of individual α‐syn molecules or subdomains. Moreover, we used cryo‐EM to explore structural alternations in α‐syn fibrils induced by two chemical compounds, EGCG and CCA, and correlated them to the potential mechanical changes. Of note, the elastic response and the local deformation events of α‐syn fibrils have little changes in the presence of the examined compounds. However, EGCG inserts into the polar groove that is formed by the N‐terminal residues of α‐syn causes an unstable fibril architecture as reflected by the attenuated rupture force. Oppositely, CCA binding to α‐syn fibrils dramatically enhances the resistance to lateral rupture forces. Overall, our work unveiled the intrinsic mechanic properties of α‐syn fibrils and their regulations by small compounds. These findings offer new insights into amyloid fibril stability and a platform for testing potential amyloid‐disrupting agents.

## 2 Results

### 2.1 Establishment of the Single‐Molecule Assay for Fibril Measurements

**Figure 1:**
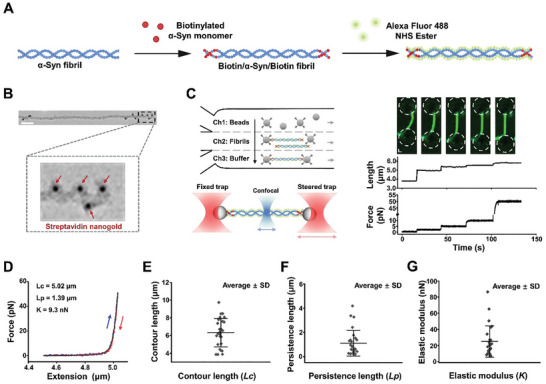
Single‐molecule assay for α‐syn fibril measurements. A) A schematic of the biotinylated α‐syn fibril assembly. α‐syn monomers are first assembled to form the fibril, then the biotinylated α‐syn monomers are added. Finally, the fibril is labeled with Alexa Fluor 488 NHS Ester. B) Detection of streptavidin‐Gold nanoparticles binding to the biotinylated α‐syn fibril by TEM. The red arrows indicate nanoparticles. Scale bars represent 100 nm. C) A diagram showing the single‐molecule experimental procedure (left). A single fibril tether is formed in channels 1–3 containing streptavidin‐coated microspheres, biotinylated α‐syn fibril, and buffer, respectively. The fibril was suspended by two streptavidin‐coated beads manipulated by two optical traps. Meanwhile, confocal lasers repeatedly scanned along the fibril. The fluorescence image and corresponding length of the α‐syn fibrils were held at 0, 2, 5, 10, and 50 pN, respectively (right). Scale bars, 1 µm. The force and extension are plotted as a function of time. D) Representative force–extension curves of a single α‐syn fibril during stretching (blue) and relaxation (red) within a force range of 0–50 pN. WLC fitting (gray) yields a contour length of 5.02 µm, a persistence length of 1.39 µm, and a elastic modulus of 9.3 nN. E–G. The contour length (E), persistence length (F), and the elastic modulus (G) distributions (*n* = 27). Error bars represent means ± SD.

We first sought to establish an OT‐based system that allows us to efficiently characterize the mechanical properties of the α‐syn fibril at the single‐molecule level. To manipulate a single fibril molecule via streptavidin‐coated microspheres in the OT assay, we developed a method to prepare micrometer‐long, biotinylated α‐syn fibrils. Taking advantage of the α‐syn fibril assembly by recruiting monomers exclusively at fibril ends, we mixed WT α‐syn monomers with preformed fibrils (PFFs) and shook the mixture for the 24 h spontaneous assembly. Then, the biotinylated α‐syn monomers were introduced into the system for an additional 3 h fibril extension so that the biotinylated α‐syn monomers were only supposed to be incorporated at both ends of the α‐syn fibril (**Figure** 1A). To verify that, we incubated assembled α‐syn fibrils with streptavidin‐gold nanoparticles and visualized the nanoparticle‐conjugated fibrils using TEM. As shown in Figure 1B, gold nanoparticles distributed at both ends of the examined α‐syn fibrils were spotted in the micrographs, indicating the successful construction of the hybrid fibril samples. To visualize the suspended fibril directly, we used the Alexa Fluor 488 as a probe and conjugated the dye to the fibril via an amidation reaction during fibril manipulation (Figure 1A).^[^ ^30^ ^]^

In the single‐fibril manipulation assay, we employed a microfluidic multichannel flow cell to swiftly construct a dumbbell fibril, allowing for quick switching of the fibril among different experimental conditions. A schematic presented in Figure 1C describes a typical experimental procedure.^[^ ^31^ ^]^ Briefly, after capturing two microspheres by two separated traps in Channel 1, we transported them to the fibril channel (Channel 2) and steered one of the traps around the anticipated position of the end of the flow‐stretched fibril for the microsphere attachment while keeping the other trap still; Once the tether was successfully formed, we transferred it to Channel 3 containing the buffer only, wherein a single fibril molecule connected between the two microspheres was ensured by the fluorescence signal from the confocal scanning at the fibril plane.

In our experimental configuration, optical manipulation enables the fibril with a nanometer‐size diameter to be stretched parallel to its axis, and this avoids the complications arising from the probe size and direct contact of the fibril to the surface as existing in the AFM assay.^[^ ^32^ ^]^ Meanwhile, fluorescence labeling permits the simultaneous monitoring of possible intermediate states of the fibril caused by the tensile stress. To demonstrate the success of our single‐molecule assay, we applied different forces on a single α‐syn fibril while recoding the fluorescence images of the fibril (Figure 1C). The imaging analysis revealed that the examined fibril was slightly bent under low forces of 2 and 5 pN. On the other hand, it exhibited a straight, fiber‐like structure under a force of 50 pN, indicating an intrinsic semiflexible characteristic of the α‐syn fibril.

### 2.2 Characterization of the Elasticity of α‐syn Fibril

Using the newly developed single‐molecule assay, we first sought to systemically characterize the elasticity of the α‐syn fibril through a stretching and relaxation experiment.^[^ ^31^ ^]^ In this assay, we moved the steered trap away from the fixed optical trap at a slow rate of 100 nm s^−1^ (corresponding to a force loading rate of 1–80 pN/s). After the force reached 50 pN, the fibril relaxation was performed by reversing the motion of the steered trap at the same rate. In these processes, the applied force on the fibril increased or decreased in small increments, and the structural changes in the fibrils were likely permitted if there were any. The force–extension relationships of the α‐syn fibril were built and compared in each cycle.

Within a force range of 0–50 pN, both the fibril stretching and relaxation processes proceeded smoothly, and no abnormal changes in force or extension were detected. Moreover, it was noteworthy that the force–extension curves overlapped perfectly for a single fibril, even after multiple cycles of stretching and relaxation (Figure 1D; Figure S1, Supporting Information). These data suggest that the α‐syn fibrils are relatively stable macromolecules, and the applied axial force within this region possibly had no pronounced effect on the fibrillar structure. We next tried to quantitatively describe the elasticity of the fibril using the worm‐like chain (WLC) model, which allows us to determine the contour length (*Lc*)‐the length following the external contour or shape of a fibril, the persistence length (*Lp*)‐the distance, beyond which a fibril starts to show curvature and the axial elastic modulus (*K*)‐the resistance of a fibril to deformation when subjected to external stress. Given that the multiple binding sites of the fibril end to the microsphere lead to the constrain on the rotation and that the α‐syn fibril is likely a semiflexible filament, the measured force–extension curves of the fibril were fitted to the following equation that can better describe the elasticity of a filament with clamped ends (no rotation) and *Lp* comparable to *Lc*.^[^ ^33^, ^34^ ^]^:

(1) $$F\;=\frac{K_{B}T}{\mathrm{Lp}}\;*\left(\frac{1}{16*{\left(1-\lambda_{u}X_{o}/\mathrm{Lc}\right)}^{2}}\right)*\left(\frac{\mathrm{Lc}/\mathrm{Lp}-24*\left(1-\lambda_{u}X_{o}/\mathrm{Lc}\right)}{\mathrm{Lc}/\mathrm{Lp}-8*\left(1-\lambda_{u}X_{o}/\mathrm{Lc}\right)}\right)$$

(2) $$\lambda_{e}=\frac{\mathrm{Lc}}{X_{o}}\;*\frac{F}{K}+1=\frac{\lambda }{\lambda_{u}}=\frac{X/X_{o}}{\lambda_{u}}\;$$

wherein *F* is the axial force acting on the fibril; *k_B_T* is Boltzmann's constant times absolute temperature; *λ* is the total fibril stretch, which is decomposed into *λ_u_*, the stretch due to the reduction in thermal fluctuations of the fibril, and *λ_e_*, the stretch due to direct axial extension; X is the current end‐to‐end distance, X_o_ is the initial end‐to‐end distance; In line with the TEM images, the fitting generated a few micrometers of *Lc* (Figure 1E). Moreover, the persistent length *Lp* is widely distributed and on the same order of magnitude as *Lc* (Figure 1F), and the axial elastic modulus is on the order of nanonewtons. In consistence with the fluorescence images, these findings reflect the rigid, straight, and heterogeneous nature of the α‐syn fibril (Figure 1C,G). The combination of mechanical manipulation and fluorescence imaging provides a direct approach for quantitatively determining the elastic properties of diseases‐associated amyloid fibrils.

### 2.3 Deformation and Disruption of α‐syn Fibril by an Axial Force

**Figure 2:**
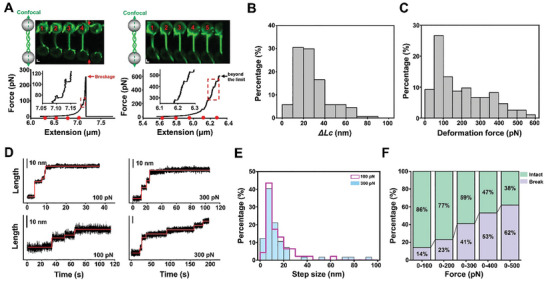
Denaturing and disruption of α‐syn fibrils by axial force. A) Stretching of α‐syn fibrils by OT at high forces with simultaneous fluorescence imaging. The fibrils are stretched until the tether breaks (left, red arrow) or reaches the maximal force of the setup (right, black arrow). The dashed boxes represent the denaturation of the fibrils during stretching. Insets: zoom in on the boxed regions. The red dots correspond to the fibril's length in the fluorescent image. Scale bars,1 µm. B) A histogram displaying the contour length change of α‐syn fibrils during all identifiable deformation events (*n* = 195). C) A histogram illustrating the deformation force distribution for α‐syn fibrils during stretching (*n* = 195). D) Representative length trajectories of α‐syn fibrils under a constant force of 100 (left) or 300 pN (right). Scale bars, 10 nm. E) The distributions of the stepping lengths detected under 100 pN (*n* = 46) and 300 pN (*n* = 57). F) The percentages of disrupted and intact fibrils in stretching experiments throughout the force range from 100 to 500 pN (*n* = 79).

Since the α‐syn fibrillar structure mostly remained intact under an axial force lower than 50 pN, we next asked under what strength this structure could be deformed or even disrupted. To this end, we further ramped up the force on a single α‐syn fibril from 50 to 500 pN. To our surprise, sudden force‐dropping events were immediately monitored in the stretching experiment within this force range, followed by continuous fibril stretching. These findings indicate that the fibrils were somehow elongated due to the tensile stretch (**Figure** **2A**). The statistical analysis revealed that changes in the contour length within these events, *ΔLc*, were predominantly concentrated on the order of tens of nanometers (Figure 2B). The distribution of forces for deformation is widespread (Figure 2C). To further corroborate the force‐induced fibril elongation, the fibrils were subjected to a constant force of 100 or 300 pN to observe the fibril length increase directly. In this condition, stepwise increases in the fibril length were monitored as expected (Figure 2D). Consistent with the stretching experiment, the step changes are mainly distributed within a ten‐of‐nanometer range (Figure 2E). Moreover, the force‐dropping and the length‐stepping events were unnoticeable from the fluorescence images, indicating subtle regional alterations in the fibril structure (Figure S2, Supporting Information). It is also noteworthy that the elongated fibril could not return to its original state after the relaxation (Figure S3, Supporting Information). These findings favor a model where the axial force causes the unfolding of α‐syn monomers within the fibril that contributes to the detected changes. In supporting that, unfolding a single α‐syn monomer was previously found to increase the molecular length by 10–50 nm, though the required force is lower.^[^ ^35^ ^]^ The discrepancy in unfolding force may be attributable to the highly stable cross‐β structure contained in the α‐syn fibril that strengthens single α‐syn monomers within the fibril (see discussion).

In addition to the length extension, the tether break in the fibril stretching experiments was recorded throughout the force range from 50 to 500 pN (Figure 2A,F). Examining the fluorescence images of the broken tether confirms that the break results from the rupture of the fibrils instead of the detachment of the biotin‐streptavidin linkage (Figure S4, Supporting Information). The fraction of the broken fibrils increased with the force, and only 38% of them can sustain a force of 500 pN (Figure 2F). Notably, some examined fibrils can even tolerate a force of up to 700 pN (the maximal force applied), reflecting the high heterogeneity of the mechanical properties of the α‐syn fibrils (Figure S5, Supporting Information).

Next, we performed the stretching experiments with α‐syn fibrils at faster rates of 0.5 and 1.0 µm ^−1^s, corresponding to maximal loading rates of–190 and 450 pN/s, respectively. According to the Bell‐Evans model, the rupture force is supposed to increase with the loading rate.^[^ ^36^, ^37^ ^]^ Indeed, an increased loading rate led to more sustained α‐syn fibrils under a high force region (Figure S6A, Supporting Information). The mild increase in the rupture force could be possibly due to the insufficient change in the loading rate. Consistently, the deformation forces detected also distribute widely under different loading rates (Figure S6B, Supporting Information). We also carried out the stretching experiments with α‐syn fibrils under a higher temperature of 37 °C. Our results demonstrated that both the rupture and deformation forces at 37 °C largely resemble those detected at 29 °C (Figure S7, Supporting Information). This finding can be reasoned by a higher heat‐denaturing temperature of 60–100 °C for α‐syn fibril as previously reported.^[^ ^38^ ^]^

Collectively, this data set provides evidence that the α‐syn fibril can be denatured and even disrupted by an axial force. The force‐induced structural alternations cause a slight fibril elongation but not the fibril breakage, which often requires a higher force.

### 2.4 EGCG Inserts Into the N‐Terminal Polar Groove and Destabilizes α‐syn Fibril

**Figure 3:**
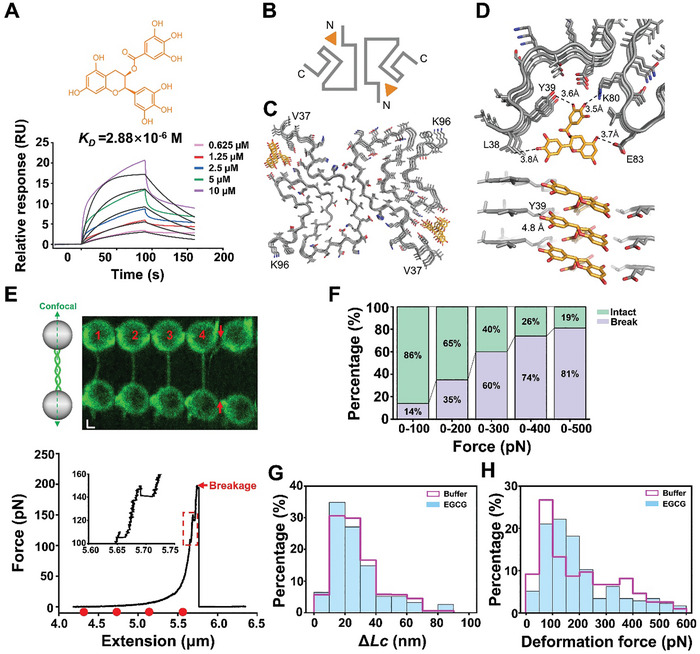
EGCG inserts into the N‐terminal polar groove and destabilizes α‐syn fibril. A) The chemical structure of EGCG and the binding affinities (*K_D_*) of EGCG to α‐syn PFFs are calculated based on the SPR association and dissociation curves. B) The topology diagram of α‐syn^EGCG^ fibril structure. The orange triangle symbol illustrates the binding sites of EGCG. C) Cartoon representations reveal the top views of three layers of the α‐syn^EGCG^ fibril. α‐Syn is colored in gray. EGCG is presented as orange sticks. The first and the last amino acid residues of the fibril core are labeled. D) The detailed top view illustrates the interactions between EGCG and the binding‐pocket residues of the α‐syn fibril (top), while the enlarged side view highlights the interactions between EGCG and the backbone amide of the α‐syn fibril (bottom). Key interaction residues and distances (Å) are labeled. E) Representative fluorescence images of α‐syn fibrils and their corresponding stretching forces as a function of length after incubation in EGCG for 2 h. Scale bars, 1 µm. The dashed boxes represent the denaturation of the fibrils during stretching. Insets: zoomed‐in of the boxed region. The red dots correspond to the fibril's length in the fluorescent image. F) The percentages of the broken and intact fibrils in the stretching experiments throughout the force range from 100 to 500 pN after the EGCG treatment (*n* = 99). G–H. The distribution of the length change (G) and the deformation force distribution (H) under EGCG treatment (light blue) (*n* = 176) compared with the ones without EGCG (pink) (*n* = 195).

Next, we used the OT and cryo‐EM to examine how chemical compounds influence α‐syn fibril structure and mechanical properties at the single‐molecule level. We first selected EGCG, which was previously reported to bind and destabilize α‐syn fibril.^[^ ^26^ ^]^ Using surface plasmon resonance, we confirmed that EGCG binds to α‐syn fibril with the binding affinity (*K_D_*) of 2.88 × 10^−6^ m (**Figure** **3A**). Furthermore, we observed that incubation of EGCG gradually dissolves α‐syn fibril in a timescale of 12 h (Figure S8, Supporting Information), confirming that EGCG can bind to α‐syn fibril and gradually disassociate matured fibrils.

To explore the structural basis of α‐syn fibril–EGCG interaction, we mixed EGCG with α‐syn fibrils and incubated the mixture for 2 h. After incubation, we froze the fibril sample for cryo‐EM study. We collected cryo‐EM images on a 300‐kV Titan Krios microscope. 115334 fibrils picked from 2497 micrographs were used to reconstruct the EGCG‐α‐syn fibril (α‐syn^EGCG^ fibril). After helical reconstruction of datasets using RELION, we obtained a 3D density map of the α‐syn^EGCG^ fibril at resolutions of 3.1 Å (Figure S9A and Table S1, Supporting Information).

The overall architecture of the α‐syn^EGCG^ fibril is similar to that of the apo α‐syn fibril. Compared to the apo fibril, two extra densities alongside the fibril core were revealed (Figure 3B; Figure S9B, Supporting Information). We next built the structure model for the α‐syn^EGCG^ fibril. Remarkably, rather than binding the interface or pocket alongside the fibril core as commonly observed in the structures of α‐syn fibril in complexed with different chemical binders,^[^ ^29^ ^]^ EGCG inserts into a shallow and polar groove (Figure 3C) formed by residues 39–46 and 80–83, which is close to the N‐terminal of the α‐syn fibril core (Figure 3C). Hydroxyl of the EGCG galloyl ring forms polar and hydrogen‐bond interaction with Lys80 and Tyr39 residues, while the hydroxyl from pyrogallol ring hydrogen bonds with Leu38, and hydroxyl from benzenediol ring hydrogen bonds with Glu83 (Figure 3D). Therefore, EGCG may interrupt the salt bridge between Glu46 and Lys80, which is pivotal for the stabilization of the Greek‐key‐like conformation in the apo α‐syn fibril (Figure 3D). Furthermore, EGCG forms a hydrogen bond with the backbone amide of Tyr39, subsequently perturbing the interlayer hydrogen‐bond network within the fibril structure (Figure 3D). Thus, insertion of EGCG into the polar groove may prise off the protofilament and destabilize the entire fibril structure.

We next explored the impact of EGCG on α‐syn by the OT assay. First, we added 50 µm EGCG to the fibrils (1 µm α‐syn monomer equivalent). After a 2 h incubation, we performed the fibril‐stretching experiment (Figure 3E). Strikingly, we observed that the presence of EGCG significantly decreased the stability of the fibrils compared to those without EGCG treatment. Specifically, 81% of the fibrils broke when the force reached 500 pN, a 19% increase compared to those without EGCG treatment (Figure 3F). This result suggests that even though there is a dose effect of EGCG, the fibrils are not destroyed after EGGC treatment for 2 h, and the stability of the fibrils has been affected (Figure 3F). Next, we asked whether this weakened stability results from more dramatic fibril deformations. Unexpectedly, the statistical analysis revealed that the *ΔLc* were still concentrated within tens of nanometers, and the deformation forces were comparable to the results obtained without EGCG (Figure 3G,H). Therefore, EGCG binding does not promote α‐syn fibril deformation. Together, our results show that EGCG can insert into the polar groove in the N‐terminal and destabilize the fibril core structure, which leads to the gradual disassembly of α‐syn fibrils.

### 2.5 CCA Binding Dramatically Enhances α‐syn Fibril Resistance to Disruptive Forces

**Figure 4:**
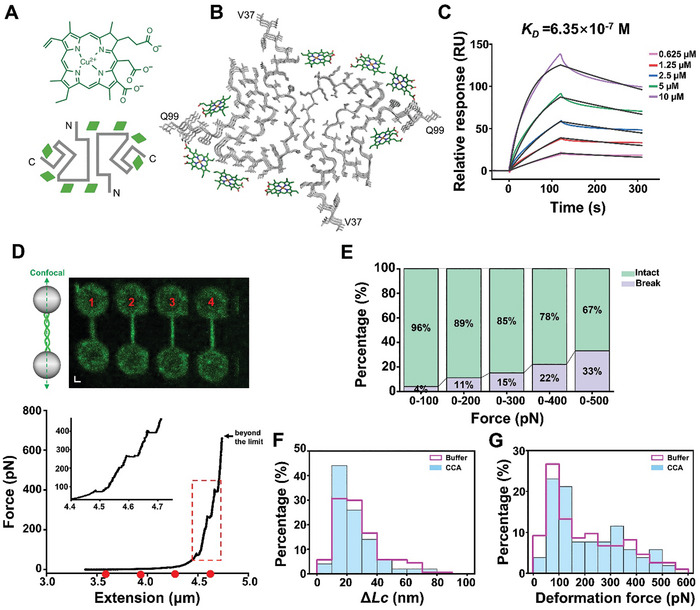
CCA binding dramatically enhances the resistance of α‐syn fibrils to disruptive forces. A) The chemical structure of CCA and topology diagram of α‐syn^CCA^ fibril structure were reported previously.^[^ ^29^ ^]^ The green rhombus symbol illustrates the binding sites of CCA. B) Cartoon representations display top views of three layers of the α‐syn^CCA^ fibril. α‐Syn is colored in gray. CCA is highlighted in green. The first and the last amino acid residues of the fibril core are labeled. C) The binding affinities (*K_D_*) of CCA to α‐syn PFFs were calculated based on the SPR association and dissociation curves. D) Representative fluorescence images of an α‐syn fibril and the corresponding force–extension curve after incubation in CCA for 2 h. Scale bars, 1 µm. The dashed boxes represent the denaturation of the fibrils during stretching. Insets: Zoomed‐in of the boxed region. The red dots correspond to the fibril's length in the fluorescent image. E) The percentages of the broken and intact fibrils in the stretching experiments throughout the force range from 100 to 500 pN after CCA treatment (*n* = 27). F‐G. The distributions of the counter length change (F) and the deformation force (G) under CCA treatment (light blue) (*n* = 52) compared with the ones without CCA (pink) (*n* = 195).

We next asked whether chemical compounds binding to different interfaces of α‐syn fibril may cause distinct effects on α‐syn fibril stability. In this case, we chose CCA, which we previously revealed to bind α‐syn at four different binding interfaces^[^ ^29^ ^]^ distinct from the one targeted by EGCG (Figure 3B). The CCA binding interfaces include the C‐pocket near the fibril core's carboxy terminus (residues 85–97), the pocket at the protofilament interface (residues 43–50 and 57–59), and a back surface near the amino terminus (residues 60–67) (**Figure** **4A,B**). CCA binds to α‐syn fibril with the binding affinity (*K_D_*) of 6.35 × 10^−7^ m, which is 5‐fold higher than EGCG (Figure 4C).

Then, we explored whether the binding of CCA may influence the mechanical properties of α‐syn fibrils at the single‐fibril level (Figure 4D). Notably, CCA significantly enhances the fibril stability according to the OT assay. Specifically, in the presence of CCA, two‐thirds of α‐syn fibrils withstand forces greater than 500 pN (Figure 4E). In sharp contrast, only 38% of apo α‐syn fibril can sustain an axial force of 500 pN (Figure 2F). However, the statistical analysis revealed that *ΔLc* and deformation force in CCA‐treated fibril was not significantly different from the apo fibril (Figure 4F,G). Thus, our results show that CCA binds α‐syn fibril and dramatically increases the resistance of the fibril to disruptive forces. It also suggests that the increased stability originates from greater stabilization between the fibril layers due to the incorporation of CCA.

## 3 Discussion

Amyloid fibrils, formed by various amyloid proteins under disease conditions, display a range of pathological behaviors.^[^ ^39^, ^40^, ^41^, ^42^ ^]^ Different from the original amyloid protein structure, these fibrils possess distinctive mechanical characteristics like rigidity and elastic modulus, which are important for fulfilling their pathological functions.^[^ ^16^, ^43^, ^44^, ^45^, ^46^ ^]^ For instance, the individual amyloid fibril formed by β_2_‐microglobulin (β_2_m) was observed by cryo‐EM to mechanically disrupt cell membranes, causing cell shape changes.^[^ ^47^, ^48^ ^]^ The Piezo1 ion channel in microglia can respond to Aβ fibril by sensing its stiffness to enhance the microglia's ability to engulf the fibril.^[^ ^49^ ^]^ Hence, understanding these mechanical properties is not only pivotal for unraveling the fundamental nature of amyloid fibrils but also holds potential for devising therapeutic strategies targeting amyloid‐related diseases.^[^ ^43^ ^]^ Our newly developed OT‐based technique allowed for an in‐depth analysis of the mechanical properties of α‐syn fibril at a single‐fibril level.

Protein fibrils often have widely varied persistent lengths, typically divided into two classes: straight fibrils with a persistence length comparable to the counter length and flexible worm‐like fibrils with a persistence length of 10–90 nm.^[^ ^50^ ^]^ The micrometer‐long persistence length obtained in our study suggests that the α‐syn fibrils are rigid, straight biopolymers that cannot be easily bent over. The extensibility of α‐syn fibrils is also limited, as reflected by the high axial elastic modulus. The persistence lengths α‐syn fibrils derived from optical tweezers experiments is an order of magnitude lower than those obtained using AFM (Table S2, Supporting Information).^[^ ^15^ ^]^ This divergence could be due to the distinct experimental conditions and the inherent differences in the measurement techniques. In AFM studies, samples are often dried and deposited on a surface using binding ligands, and the fibril profiles are fitted to a 2D worm‐chain model. These limitations could hinder an accurate measurement of the elastic properties of the fibrils. It is noteworthy that the persistent length of yeast prion fibrils measured by optical tweezers is comparable to our results.^[^ ^34^ ^]^ This congruence suggests that optical tweezers may offer a more consistent and reliable method for determining the mechanical properties of amyloid fibrils.

Even though α‐syn fibrils can be generally considered mechanically strong, these structures do deform and even beak under an axial force. Recent studies have emphasized that in macromolecular systems, arrays of hydrogen bonds can be as robust as covalent bonds, necessitating the simultaneous breaks of all hydrogen bonds to separate peptide‐peptide complexes. Our OT assays reported an axial force higher than 50 pN required for the deformation and disruption of α‐syn fibrils. Notably, the step sizes during the deformation of α‐syn fibrils are typically ≈20 nm, which falls into the range of length change in the unfolding of single α‐syn monomers.^[^ ^35^ ^]^ Considering the undetectable fluorescence signal during the sudden length change, these findings favor the force‐induced local unfolding of individual monomers or subdomains within α‐syn fibril. The protein unfolding may occur only to one of the two monomers within one layer. In this case, the global fibril structure is not completely compromised, and the fibril fracture is avoided. Compared to tens of pN of forces required to unfold a single α‐syn monomer, it takes hundreds of pN to deform them within the fibrils. This difference can be rationalized by the stacking of thousands of layers of monomers that help stabilize the individual incorporated monomers.^[^ ^51^, ^52^ ^]^ Similarly, protein deformation within the yeast prion fibril was also detected when subjected to a high axial force.^[^ ^53^ ^]^ Our work also presented a high degree of heterogeneity in the α‐syn fibril disruption. The rupture forces are widely distributed from 50 to 500 pN. This observed heterogeneity could stem from assembly defects in the fibrils and the inclusion of a minor fraction of structurally diverse α‐syn subunits within the fibrils. Supporting this hypothesis, we observed a small proportion of α‐syn segments exhibiting subtly unique structural characteristics from the 2D classification of our cryo‐EM dataset of α‐syn fibril. When these segments are mapped back onto the α‐syn fibril, they appear randomly distributed in different regions of each fibril (Figure S10, Supporting Information). The conformational heterogeneity of the α‐syn subunit within each fibril may contribute to the high lateral disruption heterogeneity observed from the OT assays.

**Figure 5:**
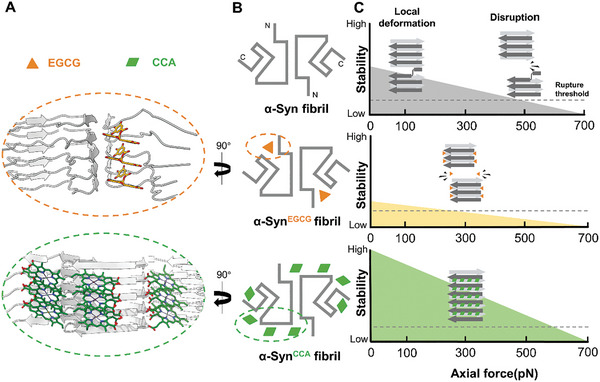
Schematic representation of the mechanical properties and structures of α‐syn fibrils and their alteration by chemical compounds. A) Side view of α‐syn^EGCG^ and α‐syn^CCA^ fibril structures showing unique interactions: EGCG inserts into the polar groove at the N‐terminus, causing fibril disassembly, whereas CCA encircles the fibril core by attaching to four different interfaces (three shown). Here, α‐syn is in gray, EGCG in orange, and CCA in green. B) Topology diagrams of α‐syn, α‐syn^EGCG^, and α‐syn^CCA^ fibril structures, highlighting the binding sites of each molecule: CCA binding sites are marked with green rhombuses, and EGCG binding sites with orange triangles. C) Correlation of the stability of α‐syn, α‐syn^EGCG^, α‐syn^CCA^ fibril and axial force. Increase axial force is linked to decreased fibril stability. The upper section depicts α‐syn subunit's local deformations within the fibril under axial force, leading to eventual disintegration. The middle part shows EGCG's role in weakening the α‐syn fibril, as indicated by a lower breaking force. Conversely, the lower section illustrates CCA binding contributes to the stabilization of the α‐syn fibril, enhancing its resistance to greater forces.

Our work also provided insights into the structural and mechanical modulation of α‐syn fibril by small molecules. We found that small molecules distinctly alter these mechanical parameters (rupture force, *ΔLc* and deformation force) by targeting different fibril interfaces. Specifically, EGCG wedges into the fibril's core from the N‐terminus, disrupting its structure and causing it to break more easily under lower forces (**Figure** **5b**). In contrast, CCA wraps around the fibril's core by attaching it to the four other interfaces, reinforcing it to withstand higher forces (Figure 5). On the other hand, the unaffected *ΔLc* and deformation force indicate that neither EGCG nor CCA significantly affects the structural deformation of monomers within the fibrils. The length of the unfolding fibrils remained comparable to that of untreated samples. No direct link between the rupture force and deformation force was evidenced for the fibrils. It is highly likely that fibril rupture does not necessarily coincide with the protein denatured region. In other words, the denatured monomer within the fibrils may not destabilize the overall stability of fibrils. Due to the technical limitations, the compound‐fibril ratio used in our cryo‐EM and OT assays in vitro is relatively high. Thus, developing advanced approaches to explore on the effects of compounds on fibril at sub‐stoichiometry will be imperative to further bridge the in vitro biophysical observation with in vivo phenomena.

As evidenced by this work, integrating cryo‐EM with our OT‐based approach enhances our comprehension of fibrils by allowing us to observe not just static structures but also the dynamic actions of individual molecules within these fibrils. This combination also facilitates the extraction of precise mechanical parameters, deepening our understanding of fibril behavior when interacting with different molecules. Such insights are crucial for the advancements in biophysics, materials science, and medical research. Overall, our work sheds light on how fibrils respond to external stretching forces in the presence of various compounds and offers a thorough perspective on the structural alterations induced by different compounds in α‐syn fibrils. This work paves the way for further exploration of structural changes in α‐syn fibrils under diverse chemical conditions at a single‐fibril level, which could be instrumental in developing small molecules aimed at managing α‐syn fibrils for disease treatment and diagnosis.

## 4 Experimental Section

### Recombinant α‐syn Monomer Expression and Purification

The human wild‐type (WT) α‐syn monomer was overexpressed and purified from *E. coli* BL21 (DE3) cells. The gene encoding human full‐length WT α‐syn was inserted into the pET‐22b+ vector. The cells were grown in LB medium at 37 °C to an OD600 of 0.8–1.2. Protein overexpression was induced by 1 mm isopropyl‐1‐thio‐D‐galactopyranoside (IPTG) at 37 °C for 4 h. The cells were collected, resuspended in 100 mm Tris‐HCl (pH 8.0) and 1 mm EDTA with 1 mm phenylmethylsulfonyl fluoride (PMSF), and lysed through a high‐pressure cell crusher. After centrifugation at 20817 × *g* at 4 °C for 30 min, the supernatant was collected and boiled for 10 min. After centrifugation, 20 mg ml^−1^ streptomycin was added to the supernatant to remove nucleic acids. After a second round of centrifugation, the pH of the supernatant was adjusted to 3.5 with 2 m HCl to allow the precipitation of other proteins. The supernatant was subsequently dialyzed in 25 mm Tris‐HCl (pH 8.0) at 4 °C overnight. After dialysis, the protein sample was purified sequentially by a Q column (GE Healthcare, 17‐5156‐01) and a Superdex 75 column (GE Healthcare, 28‐9893‐33). For the Superdex 75 column, the target protein was eluted with buffer containing 50 mm Tris‐HCl (pH 7.5) and 150 mm KCl. The purity of the protein sample was verified by SDS‒PAGE. To prepare the biotinylated α‐syn fibrils, α‐syn monomers were first labeled with EZ‐Link Sulfo‐NHS‐Biotin (Thermo Scientific, 21217), and the free biotin was removed by a desalting column (Thermo Scientific, 89882).

### Preparation of Biotinylated α‐syn Fibrils

Recombinant α‐syn monomer (200 µm, in buffer containing 50 mm Tris‐HCl, 150 mm KCl, 0.05% NaN_3_, pH 7.5) was continuously shaken at 900 rpm in a ThermoMixer (Eppendorf). After 5 days of agitation, the α‐syn fibril samples were sonicated on ice for 30 s (1 s on, 1 s off, 20% amplitude, JY92‐IIN sonicator) to obtain preformed fibrils (PFFs). To attach biotin to the fibril end, the PFFs (at a 0.5% molar ratio) were mixed with 100 µm α‐syn monomer and incubated at 37 °C with agitation at 900 rpm for 24 h. Subsequently, the biotinylated α‐syn monomers (at a 2:5 molar ratio) were introduced into the system (equivalent to 10 µm α‐syn monomer) for an additional 3‐h agitation. The free biotinylated α‐syn monomers were removed through centrifugation (20000×g, 25 °C, 1 h). The precipitate was then resuspended in PBS buffer for fluorescence labeling with a reactive dye (Alexa Fluor 488 NHS Ester, Thermo Fisher, A27034, at a 5:1 ratio) and introduced at room temperature in the dark for 1 h. Excess reactive dye was eliminated through centrifugation (20000×g, 25 °C, 1 h). Finally, the precipitate was resuspended in 50 mm Tris‐HCl, pH 7.5, and 150 mm KCl for the single‐molecule experiments.

### Negative‐Staining Transmission Electron Microscopy (NS‐TEM)

α‐Syn fibrils (10 µm α‐syn monomer) were treated with 50 µm EGCG for different time intervals (0, 3, 5, and 12 h) in 50 mm Tris‐HCl, 150 mm KCl, pH 7.5, a drop of 5 µL aliquots fibril sample were adsorbed onto a freshly glow‐discharged grid with 200 mesh carbon support film (Beijing Zhongjingkeyi Technology Co., Ltd.) for 45 s. Then, the grid was washed with 5 µL of ddH_2_O and followed by another wash with 5 µL of 3% w/v uranyl acetate. The grid was further stained with 3% (w/v) uranyl acetate for 45 s. After removing the excess buffer with filter paper, the grid was dried with an infrared lamp. TEM images were acquired on a Tecnai T12 microscope (FEI Company) operated at 120 kV.

### Streptavidin‐Gold Nanoparticles (Gold NPs) Binding to Biotinylated α‐syn Fibril

Streptavidin‐gold NP conjugate solution (Sigma Aldrich, S9059) was diluted 1:10 (v/v) in ddH_2_O and equilibrated at room temperature for 20 min. After equilibration, 50 µL of biotinylated α‐syn fibrils and 50 µL of diluted streptavidin‐gold NP solution were mixed to a final dilution of 1:20 of the streptavidin‐gold NPs, and the solutions were incubated overnight at room temperature with soft agitation. The samples were washed twice by centrifugation at 12000×g for 30 min and resuspended in ddH_2_O to remove the excess NPs. Samples were deposited onto carbon support film and stained with 3% uranyl acetate solution for their visualization by transmission electron microscopy (TEM).

### Single‐Molecule Experiments

Single‐molecule experiments were performed on an instrument combining three‐color confocal fluorescence microscopy with dual optical traps (LUMICKS C‐trap, Netherlands) in a climate‐controlled room at 23 °C. However, the temperature increased slightly to 29 ± 1 °C due to local laser trap heating. Specifically, a 1064 nm laser and a water‐immersion objective were utilized to create two orthogonally polarized optical traps, with the trap separation controlled by a piezo mirror for the beam steering of one trap. In order to accurately measure the exerted force, the trap was calibrated before attaching the polystyrene beads to the fibril. The trap stiffness was determined by measuring the effect of the optical trap on the Brownian motion of the microbeads. All of these analyses were automatically processed using the force calibration module of the software of the C‐Trap. Force measurements were carried out using back‐focal plane interferometry of the condenser top lens with a position‐sensitive detector. A computer‐controlled stage facilitated rapid movement of the optical traps within a multiple‐channel laminar flow cell, allowing for swift and complete buffer changes. Fibril molecules were trapped and held between two streptavidin‐coated polystyrene beads (4.34‐µm diameter, Spherotech) using the multichannel laminar flow cell and were tensioned by increasing the length between the optical traps, where the fibril was subjected to tension by increasing the distance between the optical traps. Then, the tethered fibril was moved to buffer channels as described for the specified assays. The typical reaction buffer contained 50 mm Tris‐HCl (pH 7.5) and 150 mm KCl. A 488 nm excitation laser was utilized to obtain fluorescence signals, and the emission was detected using a photon‐counting avalanche photodiode. The confocal pixel size was set to 75 nm, with a pixel dwell time of 0.2 ms. The interframe wait time was 20 ms for rectangular scanning.

### Single‐Molecule Data Analysis

Force extension and fluorescence data were analyzed using custom software provided by LUMICKS. Force and extension data were taken at 100 Hz. Microscope images revealed that the fibrils exhibited end‐to‐end distances comparable to their fully extended length under no externally applied forces, indicating that the persistence length (*Lp*) is of the same order as the contour length (*Lc*). Thus, a modified worm‐like chain (WLC) model was utilized to fit the force–extension data before and after the sudden drop in force. The contour length changes (*ΔLc*) were determined by comparing *Lc* before and after the discontinuities.

### Commercial Chemical Compounds

Copper chlorophyllin A (CCA, CAS no. 11006‐34‐1) and Epigallocatechin Gallate (EGCG, CAS no. 989‐51‐5) were purchased from TargetMol. CCA was dissolved in ddH_2_O (deionized distilled water) to obtain a 10 mm stock solution. EGCG was dissolved in anhydrous dimethyl sulfoxide (DMSO) to obtain a 10 mm stock solution.

### Binding Affinity Measurement by SPR

The sonicated α‐syn PFFs were transferred into PBS + Tween‐20 buffer (1.8 mm KH_2_PO_4_, 10 mm Na_2_HPO_4_, pH 7.4, 137 mm NaCl, 2.7 mm KCl and 0.05% (v:v) Tween‐20) and immobilized on a CM5 sensor chip (Cytiva). Serially diluted ligands were then flowed over the chip in PBS + Tween‐20 buffer. Binding kinetics were analyzed with Biacore evaluation software (Cytiva) to calculate the ka and kd values. The KD values were calculated as the ratio of ligand dissociation rate to association rate (kd/ka).

### Cryo‐EM Data Collection

A 4 µL sample aliquot was applied onto a glow‐discharged holey copper grid (Quantifoil R2/1, 300 mesh), then plunge‐frozen in liquid ethane using Vitrobot Mark IV (FEI, Thermo). Cryo‐EM micrographs (40 frames per micrograph) were collected on Thermo Fisher Titan Krios G4 cryo transmission electron microscope, operated at 300 kV with a BioContinuum K3 direct detector (Gatan), and a GIF Quantum energy filter (Gatan) that was used with a slit width of 20 eV. All images were automated recorded at a total dose of ≈56 electrons per Å^2^, using EPU software (Thermo Fisher Scientific).

### Imaging Processing and Helical Reconstruction

All 40 frames were aligned, summed, dose‐weighted by MotionCor2,^[^ ^54^ ^]^ and further binned to a pixel size of 0.83 Å. The defocus values of dose‐weighted micrographs were estimated by CTFFIND4.1.8.^[^ ^55^ ^]^ Helical reconstruction was performed in RELION 3.1. Fibrils were manually picked using the “Manual picking” program in RELION 3.1. The extracted segments (box size: 864 pixels, inter‐box distance of 71.7 Å) were classified by 2D classification with suitable segments selected for further processing. Initial models were generated de novo from 2D class average images using the “relion_helix_inimodel2d” program. Subsequently, 3D classification was used to optimize helical twist and rise, and 3D auto‐refinement was used. To improve the resolution of the reconstruction map, the contrast transfer function (CTF) refinement was performed. Finally, the maps were sharpened with a soft‐edge solvent mask using the standard “post‐processing” program in RELION 3.1. Overall resolution estimates were calculated based on the gold‐standard 0.143 Fourier shell correlation (FSC) between the two independently refined half‐maps. Local resolution was estimated using the Local resolution procedure in RELION 3.1 with the same mask and B‐factor in post‐processing.

### Model Building and Refinement

Based on the density map after post‐processing, the atomic models were built and modified by COOT^[^ ^56^ ^]^ using α‐syn structure (PDB entry code 6A6B) as an initial model. The model with three adjacent layers (six promoters) was refined using the real‐space refinement program in PHENIX.^[^ ^57^ ^]^ The subunit in the middle of the three layers was extracted and used as the final model.

## Conflict of interest

The authors declare no conflict of interest.

## Author Contributions

X.L. and L.B. contributed equally to this work. C.L. and B.S. conceived the project and supervised all research. X.L. and L.B. conducted all the experiments. S. Z., Q. X., W. X., Y. T., S. W., and Y. L. helped with the protein purification and single‐molecule experiments. L.B., X.L., C.L., and B.S. analyzed the data. D. L., W. L., and W. K. interpreted the data and revised the manuscript. X.L., L.B., C.L., and B.S. wrote the manuscript with inputs from all authors.

## Supporting information



Supporting Information

## Data Availability

The data that support the findings of this study are available from the corresponding authors upon reasonable request.
