# Supplementary material for: Single‐Molecule Insight Into α‐Synuclein Fibril Structure and Mechanics Modulated by Chemical Compounds
Source: Adv Sci (Weinh). 2025 Feb 14;12(14):2416721. doi: 10.1002/advs.202416721 (PMC11984887; doi:10.1002/advs.202416721)
Supplement: Supplementary file 1 — Supporting Information [file ADVS-12-2416721-s001.docx]

**Supporting Information**


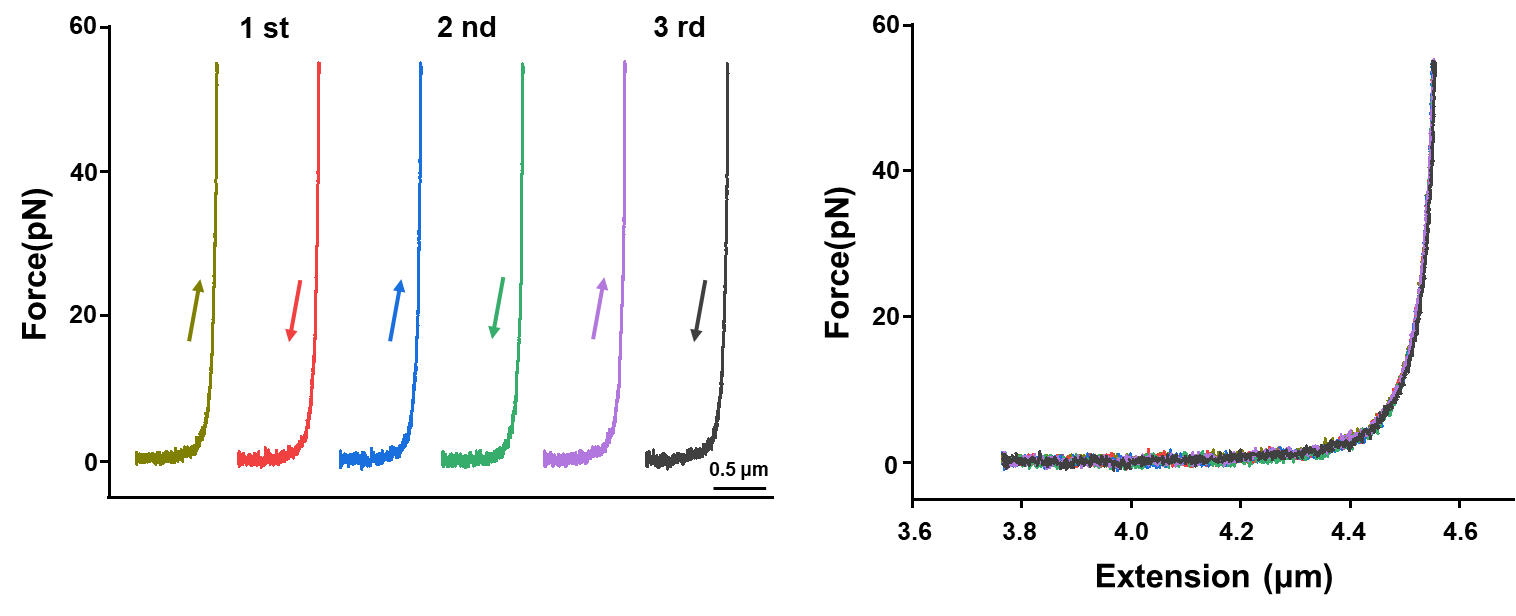


**Figure S1. The force–extension curves from multiple cycles of stretching and relaxation of a single fibril.**

The force–extension curves of a single α-syn fibril were obtained from three stretching–relaxation cycles (left). These curves overlap perfectly, suggesting that the forces within this region cause no obvious changes in α-syn fibril structures (right).

**
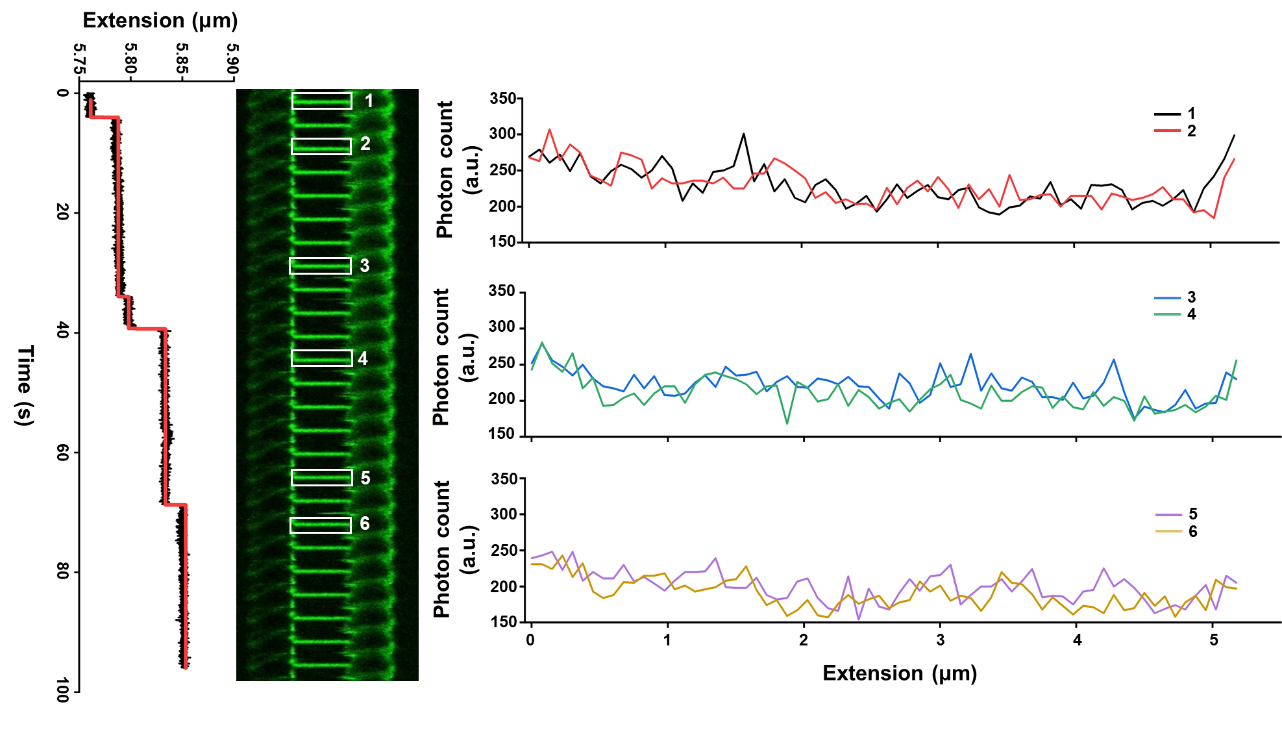
**

**Figure S2.** **Analyses on the fluorescence images of α-Syn fibril before and after elongation.**

A representative extension of a single α-syn fibril under a constant force of 100 pN and the corresponding fluorescence images are shown on the left. The fluorescence intensities along α-syn fibril within indicated individual frames are shown on the right. No obvious alternations were detected from the fluorescence images before and after α-syn fibril elongation.


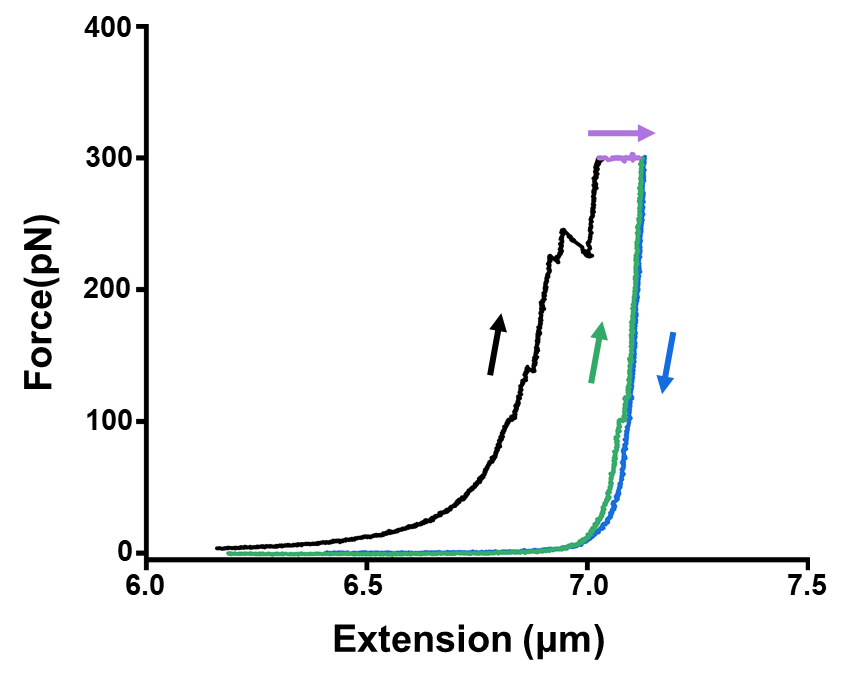


**Figure S3. The relaxation and stretching of α-Syn fibril after the elongation.**

Representative force–extension curves of a single α-Syn fibril before (black) and after its elongation. After the elongation of the fibril under constant force (purple), the elongated fibril could not return to its original state either in the relaxation (blue) or on the second stretching (green).

**
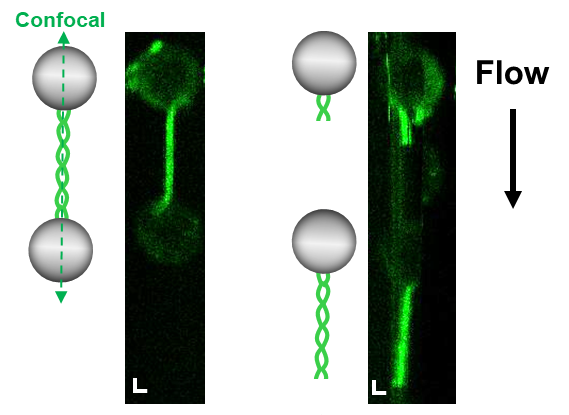
**

**Figure S4. The tether breakage results from the internal disruption of α-syn fibril.**

The fluorescence images of a broken fibril tether under a directional low show that the tether break results from the middle rupture of the fibrils. The black arrow indicates the flow direction. Scale bars, 1 μm.


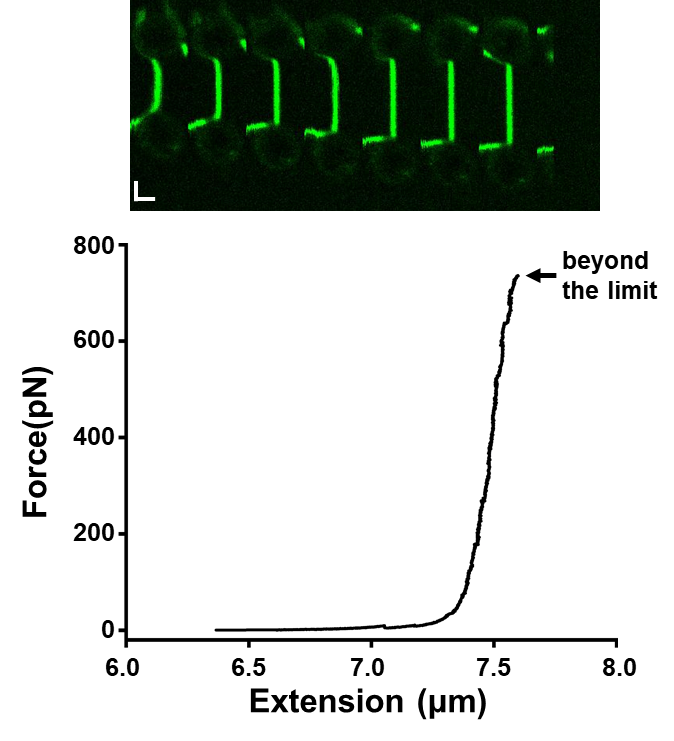


**Figure S5. A representative force–extension curve and the fluorescence images of a single α-syn fibril that sustains a disruptive force of 700 pN.**

The α-syn fibril can sustain an axial force of up to 700 pN without disruption. Scale bars, 2 μm.


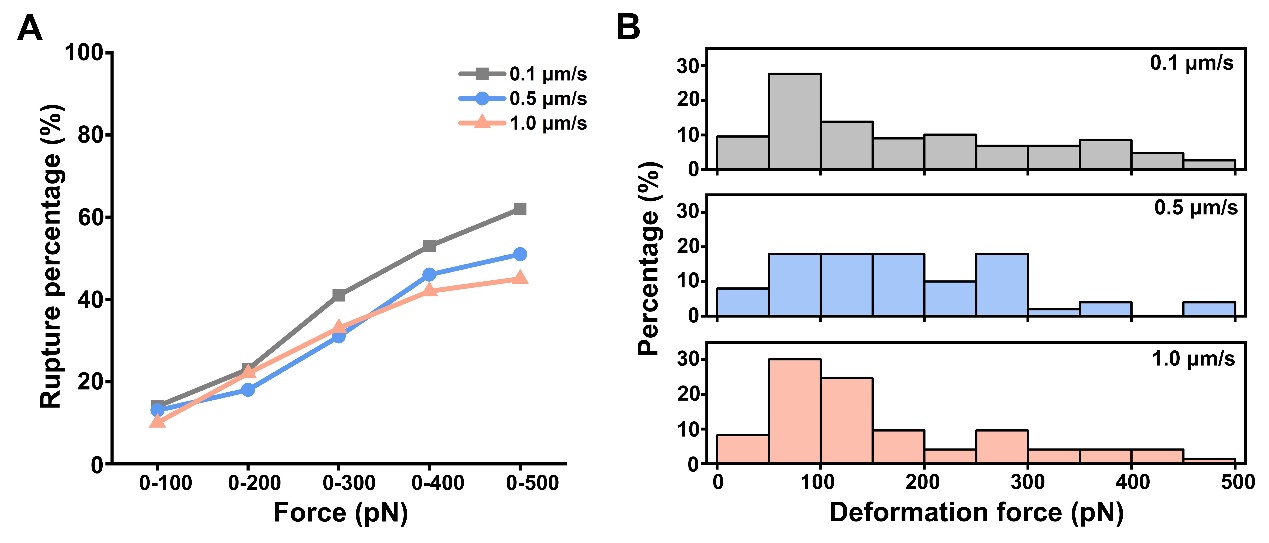


**Figure S6. Single-molecule measurements of α-syn fibrils under deferent pulling speed.**

**A.** The percentages of broken fibrils in different stretching speed experiments throughout the force range from 100 to 500 pN. This shows a clear relationship between the pulling speed and the rupture force (n = 79 for 0.1 μm/s, 39 for 0.5 μm/s, and 60 for 1.0 μm/s).

**B.** The distribution of deformation forces during the stretching process. The distribution patterns illustrate that, as pulling speed increases, the distribution tends to shift toward higher forces, reflecting an increase in the energetic accessibility of deformation pathways at faster speed (n = 188 for 0.1 μm/s, 50 for 0.5 μm/s, and 73 for 1.0 μm/s).


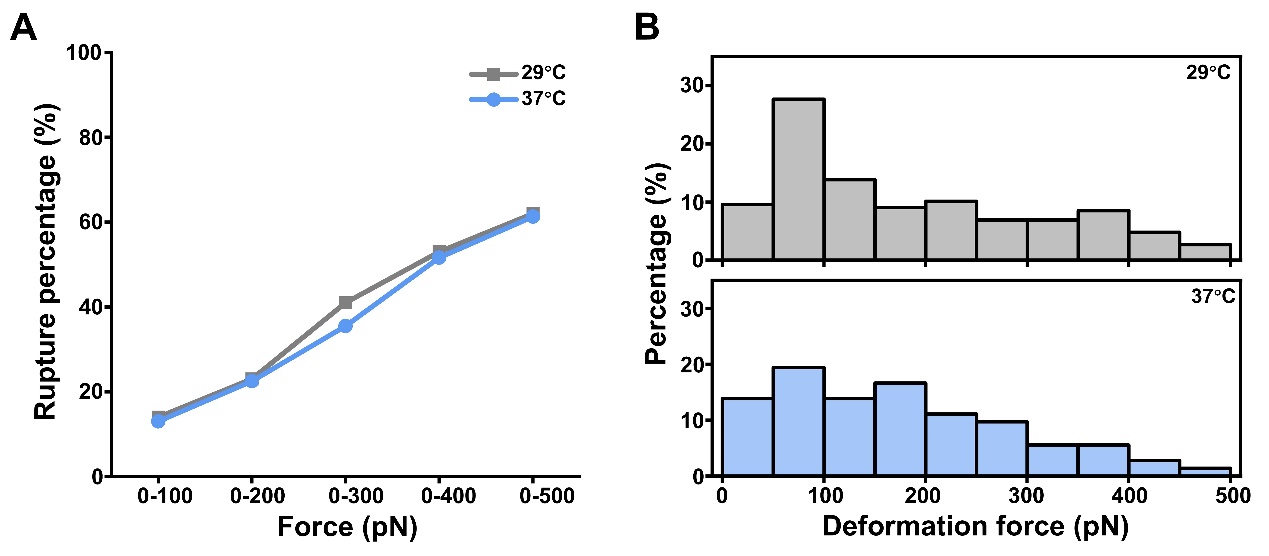


**Figure S7. Single-molecule measurements of α-syn fibrils under deferent temperatures.**

**A.** The percentage of broken fibrils in the force range of 100 to 500 pN at 29°C and 37°C, respectively. The rupture forces at 37°C are largely resemble those detected at 29°C. (n = 79 for 29°C, and 31 for 37°C).

**B.** The distribution of deformation force during stretching at different temperatures. The similar pattern in the distribution indicated that the deformation force of fibrils α-syn does not change significantly between the two temperature. (n = 188 for 29°C, and 72 for 37°C).


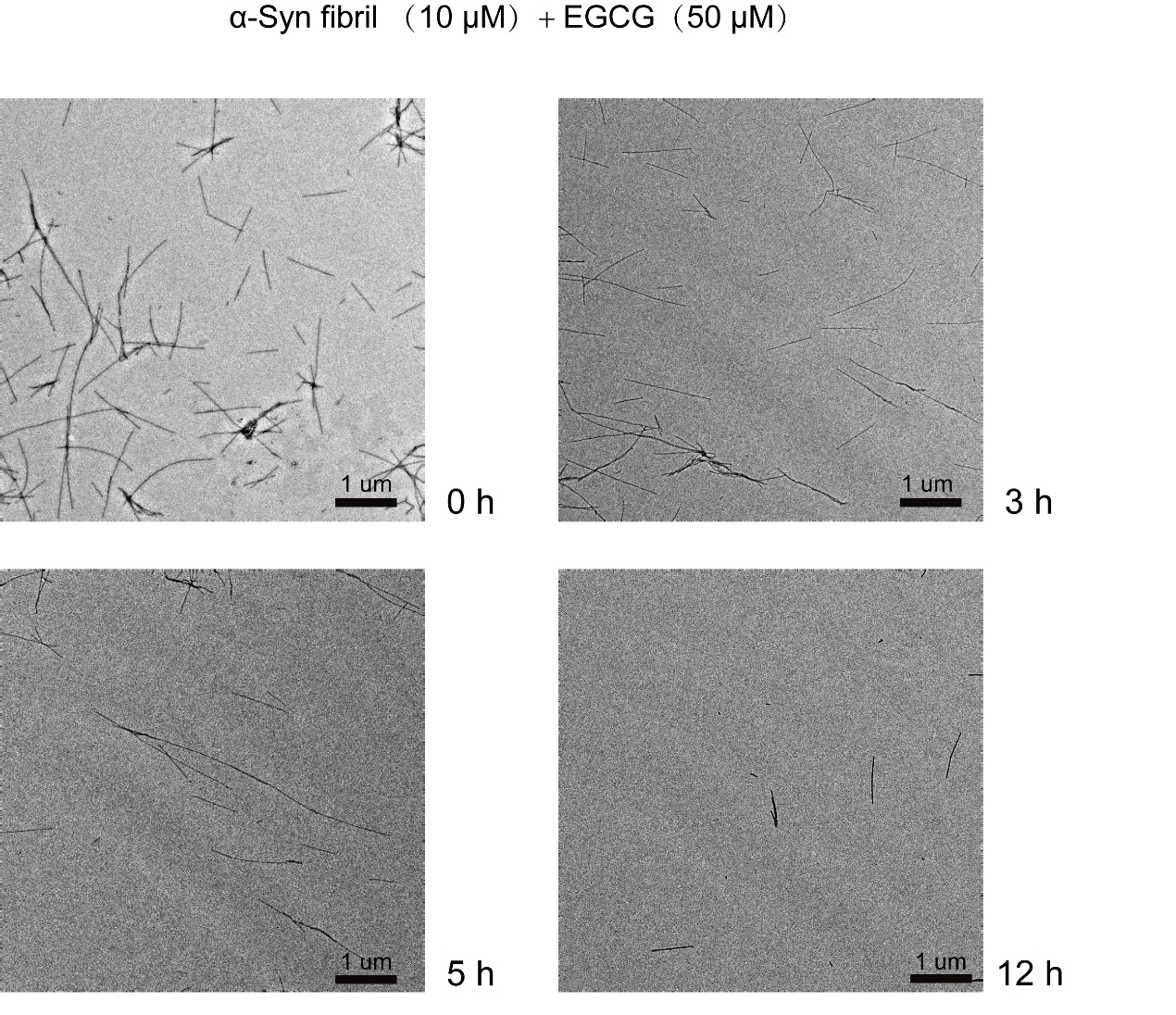


**Figure S8. TEM characterization of EGCG gradually disaggregates matured α-syn fibril.**

10 μM α-Syn fibril incubated in the presence of 50 μM EGCG for 0 h, 3 h, 5 h, 12 h. Scale bars, 1 μm.


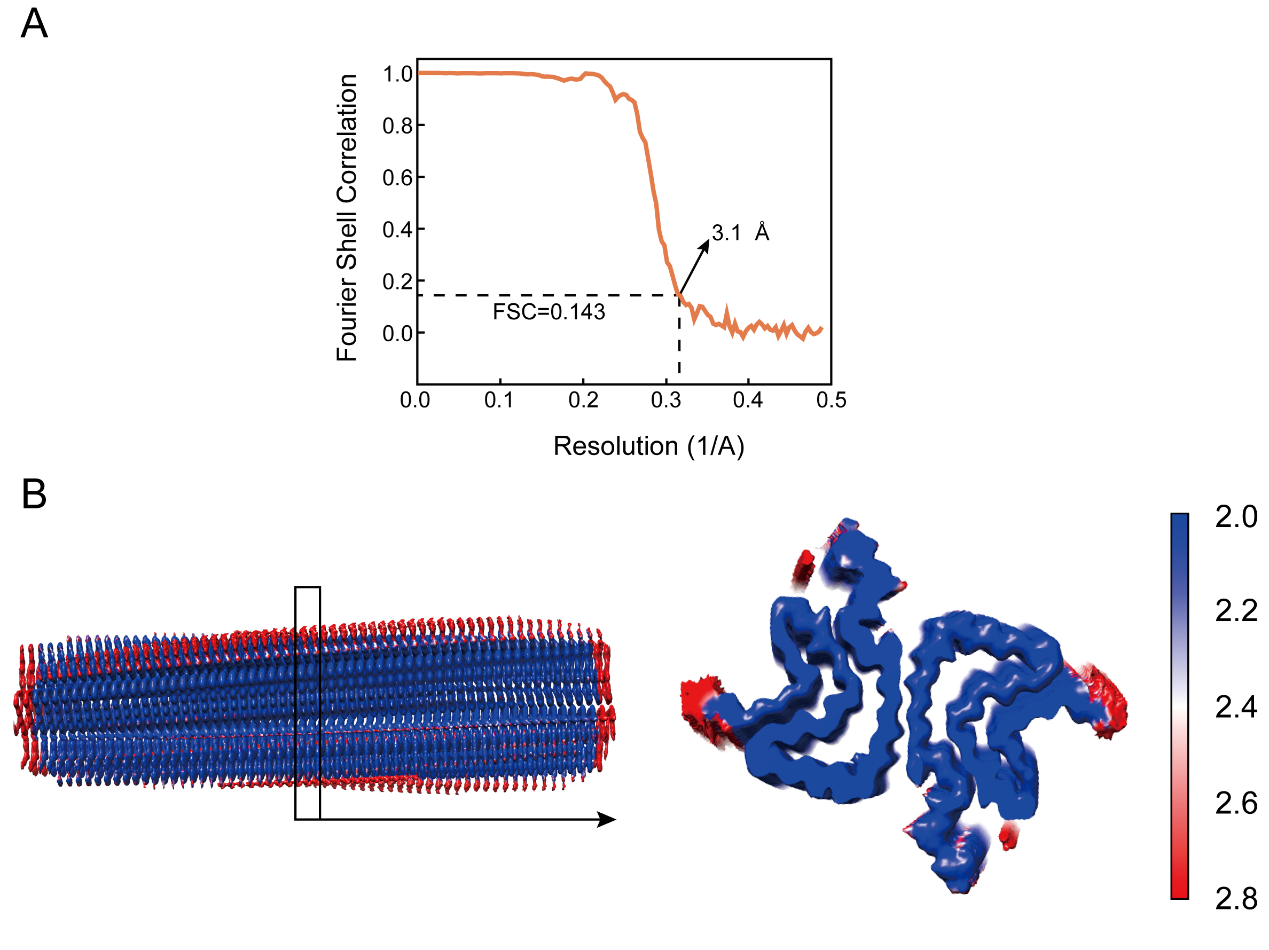


**Figure S9. Cryo-EM structure determination of the α-syn^EGCG^ fibril, related to Table S1.**

**A.** Fourier shell correlation (FSC) curve between two half maps.

**B.** Local resolution estimation. EM reconstruction maps are colored based on the local resolutions. The color scale indicating the resolution range is shown. Cross section of the central slice in the fibril is shown.


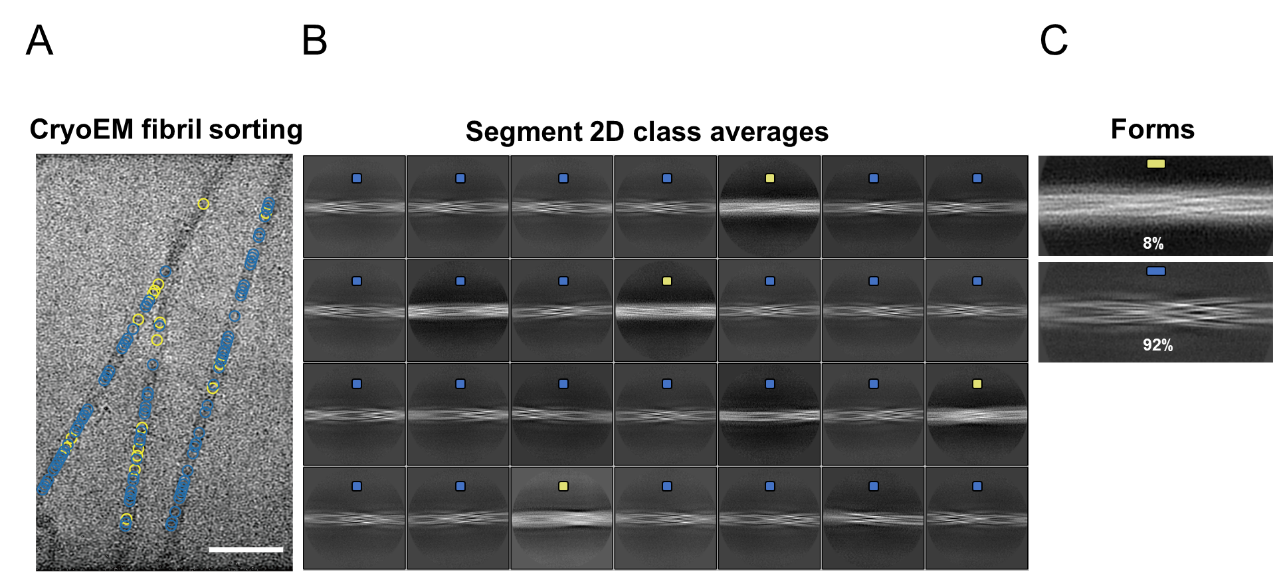


**Figure S10. Structural heterogeneity of a single fibril.**

**A.** Mapping homogeneous and heterogeneous particles back onto cryo-EM image. The scale bar (white) represents 50 nm.

**B.** The 28 most populated 2D class averages are shown for each dataset color-coded by apparent fibril features. Class averages with fibril segments showing observable crossover features are labeled in blue, and those showing ambiguous internal features are marked in yellow.

**C.** Zoomed images of selected 2D class averages from the datasets shown in (B). The percentage of each particle type was presented.

**Table S1. Statistics of cryo-EM data collection and refinement**

| **Name** | **α-Syn^EGCG^ fibril** |
| --- | --- |
| PDB ID | 8XWD |
| EMDB ID | EMD-38733 |
| **Data Collection**  Magnification |  |
|  | 105,000 |
| Pixel size (Å) | 0.83 |
| Defocus Range (μm) | -1.4 to -2.2 |
| Voltage (kV) | 300 |
| Camera | BioContinuum K3 |
| Microscope | Krios G4 |
| Exposure time (s/frame) | 0.05 |
| Number of frames | 40 |
| Total dose (e^-^/Å^2^) | 55 |
| **Reconstruction** |  |
| Micrographs | 2,497 |
| Manually picked fibrils | 115,334 |
| Box size (pixel) | 360 |
| Inter-box distance (Å) | 30 |
| Segments extracted (no.) | 1012,828 |
| Segments after Class2D (no.) | 394,384 |
| Segments after Class3D (no.) | 207,014 |
| Resolution (Å) | 3.1 |
| Map sharpening B-factor (Å^2^) | -122.284 |
| Helical rise (Å) | 4.820 |
| Helical twist (°) | -0.746 |
| **Atomic model** |  |
| Non-hydrogen atoms | 2,472 |
| Protein residues | 360 |
| Ligands | 6 |
| r.m.s.d. Bond lengths | 0.008 |
| r.m.s.d. Bond angles | 0.776 |
| All-atom clash score | 15.82 |
| Rotamer outliers | 0% |
| Ramachandran Outliers | 0% |
| Ramachandran Allowed | 15.52% |
| Ramachandran Favored | 84.48% |

**Table S2.** **Comparison of physical parameters of amyloid fibrils with previous works**

|  | **Persistence length (μm)** | **Axial elastic modulus (pN)** | | **Method** | **Notes** |
| --- | --- | --- | --- | --- | --- |
| **This work** | 1.1 | 25035 | Optical tweezers | | human α-syn |
| **Previous work** | 14.2 ± 3.3 | N.A. | AFM | | human α-syn^[1]^ |
|  | 1.5 | 5600 | Optical tweezers | | Yeast Prion^[2]^ |

**Reference**

[1] A. Makky, L. Bousset, J. Polesel-Maris, R. Melki, *Sci Rep*. 2016, 6, 37970.

[2] C. E. Castro, J. Dong, M. C. Boyce, S. Lindquist, M. J. Lang, *Biophys J*. 2011, 101, 439.
